# Ontological and Non-Ontological Resources for Associating Medical Dictionary for Regulatory Activities Terms to SNOMED Clinical Terms With Semantic Properties

**DOI:** 10.3389/fphar.2019.00975

**Published:** 2019-09-10

**Authors:** Cédric Bousquet, Julien Souvignet, Éric Sadou, Marie-Christine Jaulent, Gunnar Declerck

**Affiliations:** ^1^Laboratoire d’Informatique Médicale et d’Ingénierie des Connaissances en e-Santé, LIMICS, Sorbonne Université, Inserm, Université Paris 13, Paris, France; ^2^Unit of Public Health and Medical Informatics, University of Saint Etienne, Saint Etienne, France; ^3^EA 2223 Costech (Connaissance, Organisation et Systèmes Techniques), Centre de Recherche, Sorbonne Universités, Université de technologie de Compiègne, Compiègne, France

**Keywords:** adverse drug reaction, Medical Dictionary for Regulatory Activities, SNOMED Clinical Terms, ontology, clinical terminology, pharmacovigilance

## Abstract

**Background:** Formal definitions allow selecting terms (e.g., identifying all terms related to “Infectious disease” using the query “has causative agent organism”) and terminological reasoning (e.g., “hepatitis B” is a “hepatitis” and is an “infectious disease”). However, the standard international terminology Medical Dictionary for Regulatory Activities (MedDRA) used for coding adverse drug reactions in pharmacovigilance databases does not beneficiate from such formal definitions. Our objective was to evaluate the potential of reuse of ontological and non-ontological resources for generating such definitions for MedDRA.

**Methods:** We developed several methods that collectively allow a semiautomatic semantic enrichment of MedDRA: 1) using MedDRA-to-SNOMED Clinical Terms (SNOMED CT) mappings (available in the Unified Medical Language System metathesaurus or other mapping resources, e.g., the MedDRA preferred term “hepatitis B” is associated to the SNOMED CT concept “type B viral hepatitis”) to extract term definitions (e.g., “hepatitis B” is associated with the following properties: has finding site liver structure, has associated morphology inflammation morphology, and has causative agent hepatitis B virus); 2) using MedDRA labels and lexical/syntactic methods for automatic decomposition of complex MedDRA terms (e.g., the MedDRA systems organ class “blood and lymphatic system disorders” is decomposed in blood system disorders and lymphatic system disorders) or automatic suggestions of properties (e.g., the string “cyclic” in preferred term “cyclic neutropenia” leads to the property has clinical course cyclic).

**Results:** The Unified Medical Language System metathesaurus was the main ontological resource reusable for generating formal definitions for MedDRA terms. The non-ontological resources (another mapping resource provided by Nadkarni and Darer in 2010 and MedDRA labels) allowed defining few additional preferred terms. While the Ci4SeR tool helped the curator to define 1,935 terms by suggesting potential supplemental relations based on the parents’ and siblings’ semantic definition, defining manually all MedDRA terms remains expensive in time.

**Discussion:** Several ontological and non-ontological resources are available for associating MedDRA terms to SNOMED CT concepts with semantic properties, but providing manual definitions is still necessary. The ontology of adverse events is a possible alternative but does not cover all MedDRA terms either. Perspectives are to implement more efficient techniques to find more logical relations between SNOMED CT and MedDRA in an automated way.

## Introduction

Formal representation of semantics as provided by computational ontologies and associated semantic Web techniques have been extensively used in medical data integration systems in the last decade (Sheth et al., 2005), and they now tend to be acknowledged as a powerful means to improve the quality of the processing chain of medical data, process automatic extraction of information and knowledge from large databases or ensure semantic interoperability between disparate data processing systems (Park and Hardiker, 2009; Schriml et al., 2012; Schulz and Jansen, 2013).

In the medical domain, classic terminologies are gradually giving way to clinical terminologies, in which terms are defined using knowledge representation languages (Rossi Mori et al., 1998). An example is SNOMED Clinical Terms (SNOMED CT), a general clinical terminology whose objective is to represent all possible terms required for coding the patient record and other applications for representation of biomedical information (Khorrami et al., 2018). SNOMED CT presents several advantages compared with classic terminologies, especially the ability to apply techniques of semantic reasoning in order to build new groups of terms, whereas classic terminologies are limited to default groupings (generally made manually by experts) that are already specified as part of the terminology (Bousquet et al., 2005).

Medical Dictionary for Regulatory Activities (MedDRA) is a classic terminology used by regulatory authorities and pharmaceutical companies for coding adverse drug reactions (ADR) in pharmacovigilance databases (Brown et al., 1999). MedDRA terms are not formally defined and search is therefore limited to existing categories (Bousquet et al., 2005). It is frequently difficult to identify the exact MedDRA category that represents a given medical condition under investigation in a sufficiently specific and exhaustive way, for example, during a pharmacovigilance database search (Brown, 2003).

Since several years, we have performed studies that showed that a knowledge-based approach is efficient for building new groups of ADR terms with World Health Organization Adverse Reaction Terminology (WHO ART) (Alecu et al., 2006) (Iavindrasana et al., 2006) and with MedDRA (Henegar et al., 2006; Declerck et al., 2012; Asfari et al., 2016; Souvignet et al., 2016a) in an automated way. This means that starting from a resource containing formal definitions of ADR terms, it is possible to make queries that correspond to a case definition in order to retrieve the related set of terms. This strategy was applied in Pharmacovigilance Adverse Reaction Terminology Server (Alecu et al., 2007) where building a knowledge base for all WHO ART terms was a challenge. Indeed, all definitions were to be set manually, and we therefore focused on automated ways to enrich WHO ART (Iavindrasana et al., 2006). We found that mapping of WHO ART with SNOMED CT by means of the Unified Medical Language System (UMLS) metathesaurus proved to be a very efficient method to build formal definitions of WHO ART terms in an automated way (Alecu et al., 2008).

Difficulties we encountered for enriching WHO ART now appear at a larger scale in MedDRA due to a growing number of terms and a more complex organization of MedDRA. Indeed, only about 50% of MedDRA terms [excluding lowest level term (LLT)] were associated with a SNOMED CT concept in UMLS (Bodenreider, 2009). Therefore, the mapping method we applied to WHO ART was a fair starting point but proved to be insufficient for obtaining an exhaustive enrichment of MedDRA.

Our objective was to evaluate the potential of reuse of ontological and non-ontological resources for defining and/or enriching definitions of MedDRA terms. We present in this article several complementary methods that may benefit different levels of automation and could also be reused in order to semantically enrich other terminologies. These include methods such as i) extracting SNOMED CT definitions based on MedDRA-to-SNOMED CT mappings available in the UMLS metathesaurus or other mapping resources and ii) developing lexical and syntactic methods using MedDRA term label information. The ability to reuse the selected ontological and non-ontological resources was measured by comparing the number of MedDRA terms associated with a formal definition after processing of these resources with the number of MedDRA terms to define. Additionally, we manually curated some term definitions using expert knowledge that allowed us to evaluate the time necessary and validated a sample of the formal definitions provided by the previous methods. We stored formal definitions of MedDRA terms in a semantic resource named OntoADR (Bousquet et al., 2014).

The organization of OntoADR and results of semantic queries on OntoADR have been already published (Bousquet et al., 2014; Souvignet et al., 2016b). This article presents methods we implemented for reusing ontological and non-ontological resources to enable the formalization of the semantics and results obtained with each of these methods, how they automate the development of formal representations of MedDRA terms, the limits related to these methods, and additional developments that would be required for a more complete semantic enrichment of MedDRA.

## Background

### Hierarchical Organization of Medical Dictionary for Regulatory Activities

**Figure 1:**
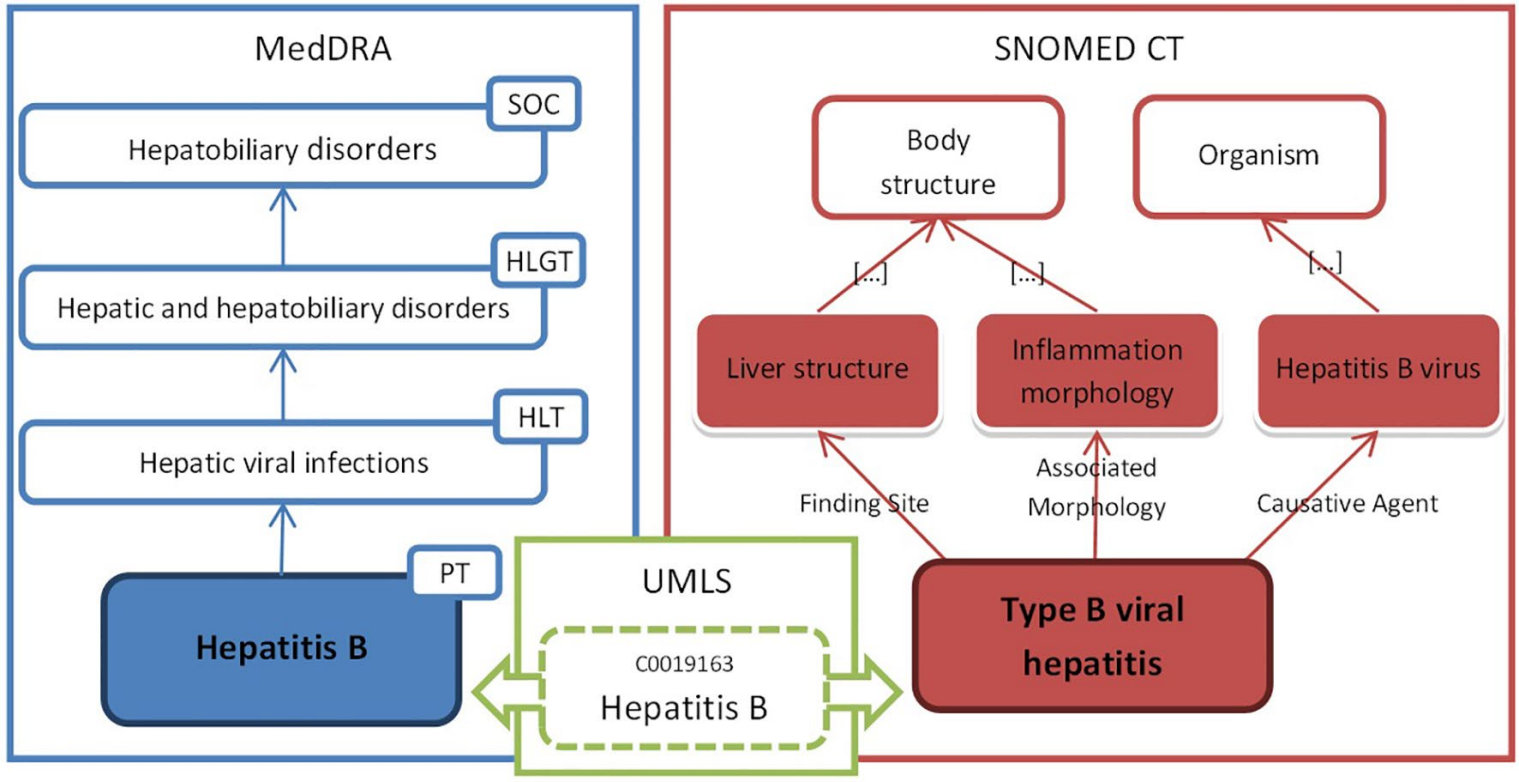
Example of the formal definition of “hepatitis B” in OntoADR.

The MedDRA hierarchy consists of five levels (from broad to narrow), among which four are depicted in **Figure 1**: System Organ Class (SOC), e.g., hepatobiliary disorders; higher level group term (HLGT), e.g., hepatic and hepatobiliary disorders; high level term (HLT), e.g., hepatic viral infections; preferred term (PT), e.g., hepatitis B; and LLT not shown on the figure. The PT level is preferred for data analysis and retrieval. MedDRA was defined as multi-axial because one PT may be present in one primary SOC and also in several secondary SOC. However, one PT may exist only within one single HLT within a SOC. As HLT within a SOC constitutes disjoint classes, it is seldom reliable to consider only one HLT or higher level category when searching for MedDRA terms related to a pharmacovigilance safety topic (Bousquet et al., 2005; Asfari et al., 2016).

Moreover, it was recognized that HLT are not always sufficient to represent clinical conditions involving several organs (e.g., anaphylactic shock involving the kidney, liver, cardiovascular, and respiratory systems) because they only group together terms belonging to the same SOC. When searching for signals associated with a drug, MedDRA terms representing the suspected ADR must thus be identified prior to the running of signal detection algorithms Souvignet et al. (2012). For instance, if one suspects a given drug to cause acute renal failure, using the MedDRA term “renal failure acute” is generally not sufficient for the algorithms to extract a signal because the acute renal failure condition can be coded with several related MedDRA terms by health professionals (e.g., “renal impairment,” “blood creatinine abnormal,” or “dialysis”). Identifying clinically related terms in MedDRA is not an easy task, as those terms might exist in different locations of the MedDRA hierarchy.

Since several years, the Maintenance and Support Services Organization, which is responsible for MedDRA maintenance and diffusion, builds standardized MedDRA queries (SMQ) to address these issues (Mozzicato, 2007). SMQs consist of sets of PT from different branches of MedDRA that allow describing a particular medical condition and are intended to aid in case identification. SMQs are a way to describe safety topics relevant for pharmacovigilance that are not covered by the HLT and HLGT present in the MedDRA hierarchy. However, the achievement of SMQ raises important difficulties.

SMQs are currently developed manually by experts from the Maintenance and Support Services Organization, which is time consuming. Furthermore, once defined, they should not be modified or customized, and the existing SMQs do not cover all issues possible with drugs. Because experts have (even slightly) different understandings of the medical condition targeted by an SMQ, the kind of terms and the rationale for their selection may differ from an SMQ to another. This means that from one group of experts to another, for the same safety topic, the list of MedDRA terms selected could be different. Because SMQs are manually implemented, they could also miss important MedDRA terms. For those different reasons, the development of methods for automated selection of MedDRA terms on the basis of semantic information is desirable. Indeed, an automation of the process of PT selection in SMQ, even partial, could increase the quality and reproducibility of the SMQ and allow an important saving of time.

### Difficulties for Searching Terms in Medical Dictionary for Regulatory Activities

The performance of pharmacovigilance systems based on spontaneous reporting is dependent on the information systems in which case reports are stored. In particular, these systems are subordinated to the ability of users to retrieve and exploit case reports in order to 1) reinforce existing knowledge on drug safety, 2) make assumptions about the existence of a causal relationship between a drug and an adverse event, and 3) evaluate the available information to implement regulatory measures to secure drug therapies. The search for pharmacovigilance case reports is difficult because it is necessary to identify the medical terms indicating the safety topic that one wishes to evaluate. In general, a term is not sufficient to designate this safety topic, and it is preferable to look for all case reports in relation to a set of terms (Hauben et al., 2006; Hansen et al., 2007). According to the MedDRA^®^ Data Retrieval and Presentation: Points To Consider (ICH Working Group, 2018), “clinically related PTs might be overlooked or not recognized as belonging together because they might be in different groupings within a single SOC or they may be located in more than one SOC.”

**Figure 2:**
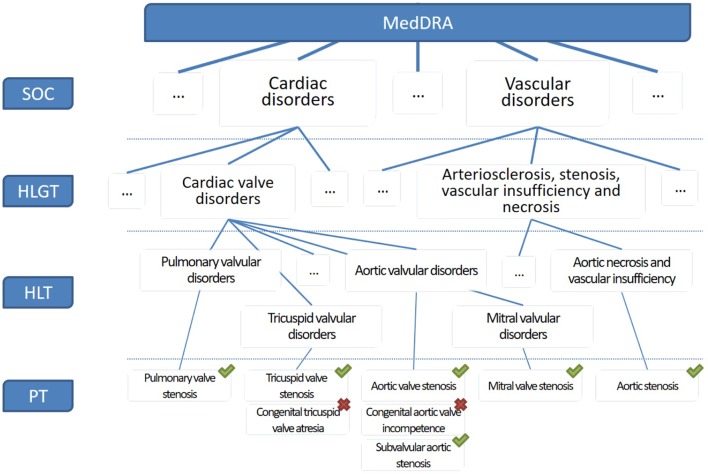
Extract of the MedDRA hierarchy showing preferred terms related or non-related to valvulopathies.

**Figure 2** shows the problem of finding terms in MedDRA. Terms associated with a green tick are related to valvulopathy, while terms marked with a red cross do not correspond to valvulopathy. It is observed that the search terms are located in different branches of the terminology, which requires the pharmacovigilant specialist more time and effort to carry out his query. In addition, several HLT or HLGT must be combined to arrive at the final result, and irrelevant terms are present in these groups, which means that a search based on HLT or HLGT groupings will be associated with a large number of irrelevant PT. Another method for searching MedDRA terms is the textual query, but the terminology seems complex and does not reveal discriminating strings in the search for valvulopathies. For example “stenos * aort *” gives as results “stenosis of the aortic valve,” “congenital aortic stenosis of the valve,” and “stenosis of the mitral valve and insufficiency of the aortic valve,” but it is necessary to perform additional text searches corresponding to findings for valvular involvement such as calcification or insufficiency.

### Interface, Aggregation, and Reference Terminologies

Schulz et al. (2017) recently evaluated how interface, aggregation, and reference terminologies may interact in the context of the new 11^th^ version of the International Classification of Diseases and its relations with SNOMED CT. Interface terminology was defined by Rosenbloom et al. (2006) as a “systematic collection of health care–related phrases (terms) that supports clinicians’ entry of patient-related information into computer programs [ … ].” According to Spackman (1997), the “main purpose of a reference terminology [ … ] is the retrieval and analysis of data.” The term “aggregation terminology” was first introduced by Rogers (2005) to designate a classification systems in which its main purpose is to enable “statistical aggregation,” further defined by Schulz et al. (2017) as consisting of single hierarchies and disjoint classes.

We consider that such clarification would be useful in our case study, to better explain what we intend to do with MedDRA and SNOMED CT. Within our approach, MedDRA is the aggregation terminology, and SNOMED CT is the reference terminology. As our purpose is to improve retrieval and not coding of MedDRA terms, we did not work on building an interface terminology. Such interface terminology would be desirable to facilitate data entry in pharmacovigilance databases but is outside of our scope. In the following paragraph, we show how a graphical user interface implementing SNOMED CT as a reference terminology could help users’ experience in selecting MedDRA terms and potentially improving search in pharmacovigilance databases.

### Rationale for Supplementing Medical Dictionary for Regulatory Activities With Formal Definitions

We consider it is possible to overcome the limitations associated with the organization of MedDRA terminology and the difficulty to identify related MedDRA terms, by proposing an alternative method for the grouping of PTs based on their medical meaning rather than their position in the hierarchy. This new method is based on PT modeling in a form that allows logical inferences by a computer. From a technical point of view, the implementation of this method is based on knowledge engineering, a branch of artificial intelligence in which it is possible to describe MedDRA terms using a formal language (Bousquet et al., 2014). In the field of knowledge engineering, we define “ontology” as the set of objects of a domain and relations between these objects.

While McKnight (1999) recognized the need for user-directed composition of controlled health terminologies, and the required improvement of the user interface in the context of data entry, we believe that such user interface is also of great importance to enable composition for data retrieval. In a previous work, we implemented OntoADR query tools, a graphical user interface that relies on OntoADR, and compared the performances of eight users in selecting MedDRA PTs with the MedDRA web browser in a pilot study on five medical conditions (Souvignet et al., 2019). Although the number of medical conditions was low, we observed a statistically significant improvement by using OntoADR query tools compared with the MedDRA web browser for selecting MedDRA PTs (+27% precision and +34% recall). Similar to Maedche and Staab (2001), we consider that the target application may serve as a measure for validating the implemented ontology and believe that such criteria is more important than criteria relying only on the evaluation of the ontology without taking into account the context where it is used. This pilot study confirmed the validity of our approach and justifies that we continue the implementation of our mappings between MedDRA and SNOMED CT.

**Figure 3:**
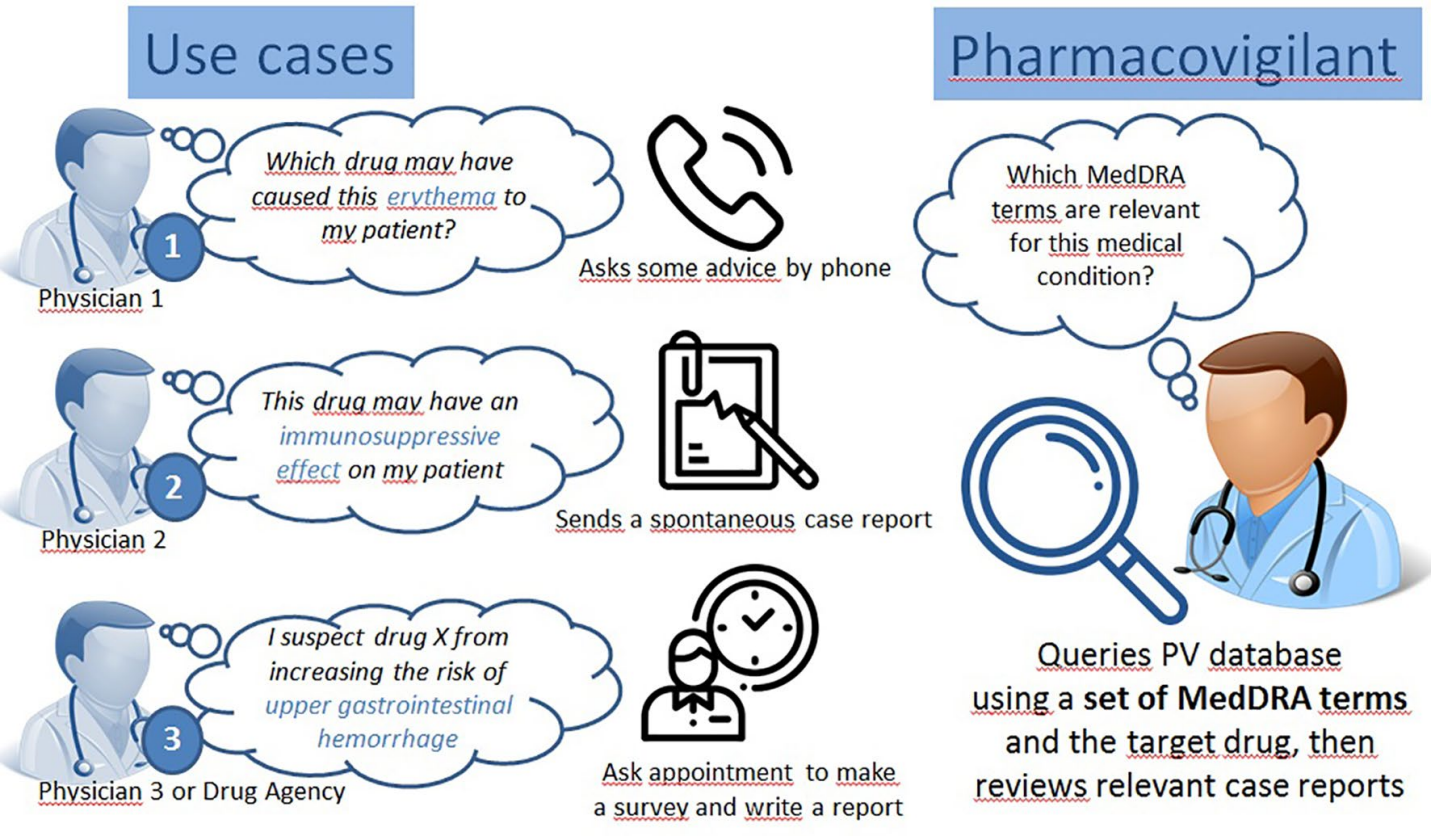
Three use case where data retrieval in a pharmacovigilance database is necessary.

**Figure 4:**
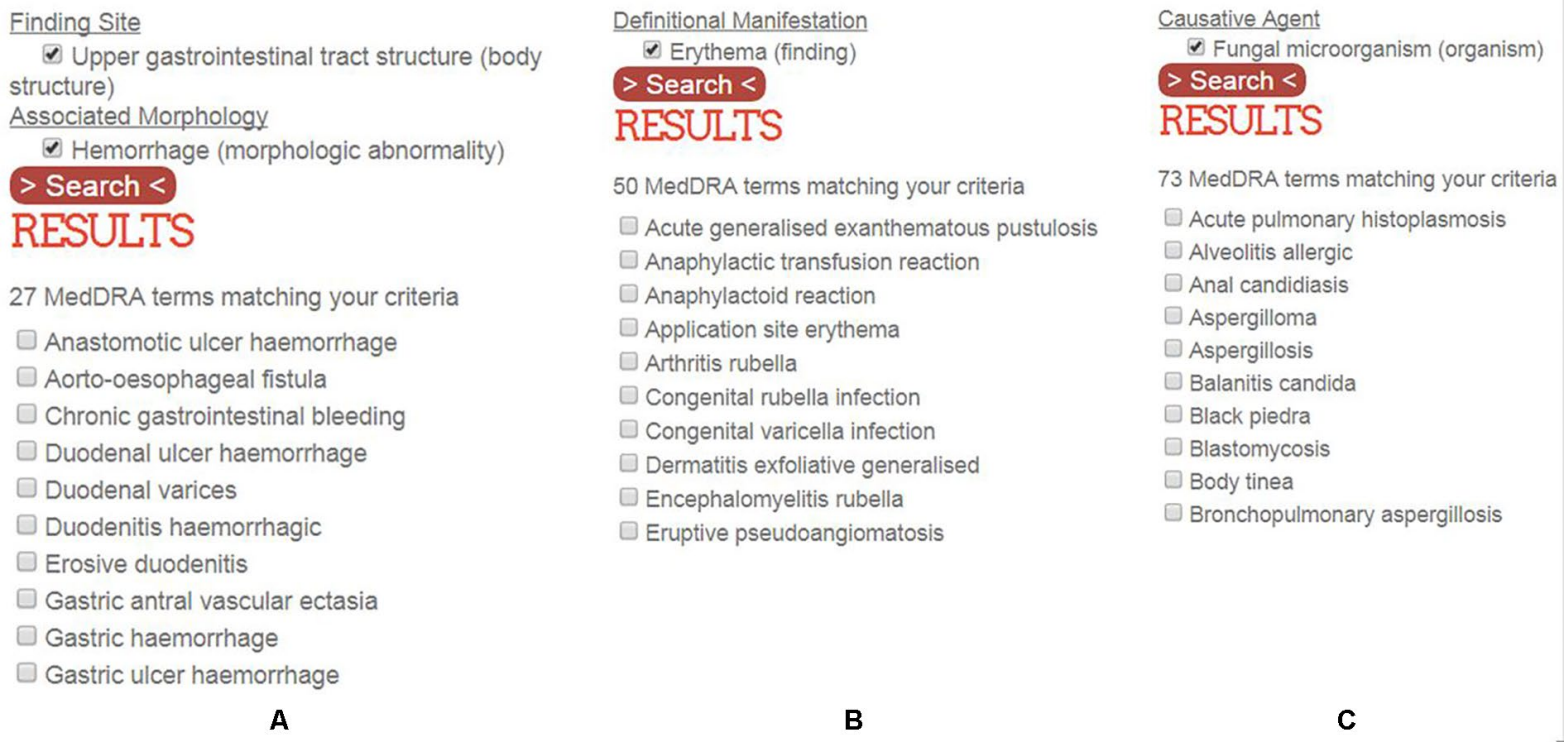
MedDRA terms associated to a formal definition (only the 10 first MedDRA terms retrieved by the query are depicted). **(A)** MedDRA terms associated to upper gastrointestinal hemorrhage, **(B)** MedDRA terms describing medical conditions associated with erythema, **(C)** MedDRA terms describing infectious diseases induced by fungi.

In order to address a safety issue, it is necessary to identify case reports in pharmacovigilance databases relative to this issue. A safety issue may concern the causality assessment of drug D in the occurrence of medical condition C. **Figure 3** shows the example of three use cases where one is evaluating the causal role of suspected drug D in three medical conditions: a) upper gastrointestinal hemorrhage, b) medical conditions with symptom of erythema, and c) fungal infectious disorders. A single MedDRA term is usually not sufficient to characterize a given medical condition. OntoADR is intended to support selection of MedDRA terms according to different criteria. **Figure 4** depicts the query performed in OntoADR to retrieve MedDRA terms associated to these medical conditions, e.g., “finding site: upper gastrointestinal tract,” and “associated morphology: hemorrhage,” and the 10 first MedDRA terms retrieved by this query, e.g., anastomotic ulcer hemorrhage, aorto-esophageal fistula, chronic gastrointestinal bleeding, etc.

**Table 1** shows parts of formal definitions for 10 MedDRA terms associated with upper gastrointestinal hemorrhage among 27, in particular, SNOMED CT concepts that are filler of relations “finding site” and “associated morphology.” Fillers that are not relevant to upper gastrointestinal hemorrhage are in italic, e.g., *Stenosis* for the MedDRA PT “anastomotic ulcer hemorrhage.” In case the filler of the “associated morphology” relation is “hemorrhage,” the query is immediately satisfied for this condition, e.g., three MedDRA terms (aorto-esophageal fistula, gastric antral vascular ectasia, and gastric hemorrhage) are defined as having “hemorrhage” as their associated morphology. When “hemorrhage” is not the filler of the “associated morphology” relation, other relevant fillers may be retrieved thanks to the subsumption mechanism that establishes hierarchical relations between a parent concept and its children concept. For example, “hemorrhage” subsumes “acute bleeding ulcer,” ‘bleeding varices,” “chronic hemorrhage,” and “hemorrhagic inflammation.” “Upper gastro intestinal tract structure” subsumes “duodenal structure,” “esophageal structure,” “gastrojejunal junction structure,” “pyloric antrum structure,” and “stomach structure.”

**Table 1:** Finding site and associated morphology of 10 MedDRA terms describing upper gastrointestinal hemorrhage among 27.

	hasFindingSite	hasAssociatedMorphology
Anastomotic ulcer haemorrhage	Gastrojejunal junction structure	Acute bleeding ulcer Stenosis
Aorto-esophageal fistula	Aortic structure Esophageal structure	Hemorrhage
Chronic gastrointestinal bleeding	Stomach structure	Chronic hemorrhage
Duodenal ulcer haemorrhage	Duodenal structure	Acute bleeding ulcer
Duodenal varices	Portal vein structure Duodenal structure	Bleeding varices
Duodenitis hemorrhagic	Duodenal structure	Hemorrhagic inflammation
Erosive duodenitis	Duodenal structure	Hemorrhagic inflammation
Gastric antral vascular ectasia	Pyloric antrum structure	Hemorrhage Angiectasia
Gastric hemorrhage	Stomach structure	Hemorrhage
Gastric ulcer hemorrhage	Stomach structure	Acute bleeding ulcer

### Semantic Enrichment

The traditional process of domain ontology construction is based on expert intervention (Bedini and Nguyen, 2007). Although this manual procedure guarantees a fair quality of the generated resource, it suffers from several difficulties; among those are the cold start problem (starting from scratch) and the lack of availability of domain experts (Qawasmeh et al., 2018). In fact, the high cost of experts’ interventions is the major bottleneck identified early in the state of the art of ontology construction (Cullen and Bryman, 1988; Simperl et al., 2006; Balakrishna et al., 2010). This bottleneck justifies reusing and linking existing resources, when available, to create new ontologies (Alani, 2006). Reuse is not always possible because ontologies may not exist in the field of interest. For example, Mazo et al. (2017) describe a histological ontology of the human cardiovascular system and report that they “did not find in the State-of-the-Art an ontology of histology neither a similar organization of hierarchies of histology terms that [they] may be able to reuse.” At the opposite, when all the ontologies that are needed are available, it is sufficient to reuse and assemble them, such as in the example of the development of the orthology ontology (Fernández-Breis et al., 2016).

Ontology enrichment is the task of extending an existing ontology with additional concepts and semantic relations and placing them at the correct position in the ontology (Petasis et al., 2011). Automatic ontological construction is often based on learning (Maedche and Staab, 2001; Buitelaar et al., 2005). Such approach can be based on unstructured texts (Asim, 2018; Cimiano et al., 2006; Emani et al., 2015; Costa et al., 2016; Dasgupta et al., 2018), informal ontologies (Astrakhantsev and Turdakov, 2013), or linked data (Gavankar et al., 2012; Tiddi et al., 2012; Riga et al., 2017). A particular case of unstructured data corresponds to the labels of ontology identifiers that may be very dense with information. Such information described as “hidden semantics” by Third (2012) received little attention, at the exception of the gene ontology identifiers (e.g., Quesada-Martínez et al., 2015). SNOMED CT identifiers may also benefit from such approach, but this was limited to taking into account the “acute” and “chronic” qualifiers for evolution of diseases (Rector and Iannone, 2012) and the occurrence of congenital diseases (van Damme et al., 2018). Such “hidden semantics” were also detected by Nadkarni and Darer (2010) to build correspondences between MedDRA and SNOMED CT, but these correspondences were not associated with relations, which makes this work interesting for reuse but requires reengineering to transform the correspondences into semantic relations.

One of the major difficulties of these approaches is the extraction of non-taxonomic relationships (Dahab et al., 2008; Sánchez and Moreno, 2008; Villaverde et al., 2009; Petasis et al., 2011; Serra et al., 2014). Furthermore, several automatic approaches for ontology reusing and engineering still require domain experts and knowledge engineers (Bobed et al., 2012). Thus, semiautomatic approaches could be a good alternative (Balakrishna et al., 2010).

Semiautomatic approaches employ intelligent methods to significantly reduce, without completely replacing, human efforts (Huang et al., 2014). In such approaches, the role of experts could be limited to validating final automatic learning results (Wächter and Schroeder, 2010) or suggesting improvements at the end of ontology life cycle (Alobaidi, 2018). Expert intervention can be achieved with the help of graphical user interface (Wächter and Schroeder, 2010), spreadsheets (Blfgeh et al., 2017; Judkins et al., 2018), or specified pipelines such as the eXtensible ontology development (He et al., 2018).

### NeON Methodology

After comparing several methods for ontology development, we selected the NeON methodology (Suárez-Figueroa et al., 2012) because it was the most appropriate to illustrate the strategy that we followed for designing OntoADR. While other methodologies may also be considered for knowledge engineering and may be more relevant in other contexts, we considered that dimensions of reuse were the most important features when selecting NeON.

While other approaches for ontology engineering provide methodological guidance, the NeON Methodology does not prescribe a rigid workflow. It suggests a variety of pathways based on nine flexible scenarios that address common issues, such as reusing, reengineering, and merging ontological resources. These ontological resources also comprise ontology design patterns (Aranguren, 2008; Gangemi, 2005; Blomqvist, 2008; Presutti and Gangemi, 2008), which are generic templates or abstract descriptions proposed to enforce best practices in ontology implementation. One particularity of NeON matching well with our specific approach is that it also takes into account reusing and reengineering of non-ontological resources, which is not the case of other methodologies such as METHONTOLOGY (Fernández-López et al., 1997) and On-To-Knowledge (Sure et al., 2004). These non-ontological resources may consist of structured data such as terminologies (Jimeno-Yepes et al., 2009) or databases, unstructured data (e.g., articles), or semi-structured data (e.g., XML, JSON) (Qawasmeh et al., 2018). In addition to these nine scenarios, NeON also integrates support activities such as knowledge acquisition, documentation, and evaluation that should be carried out during the whole ontology development cycle.

## Material and Methods

### Summary of the Method

#### Application of the NeON methodology

**Figure 5:**
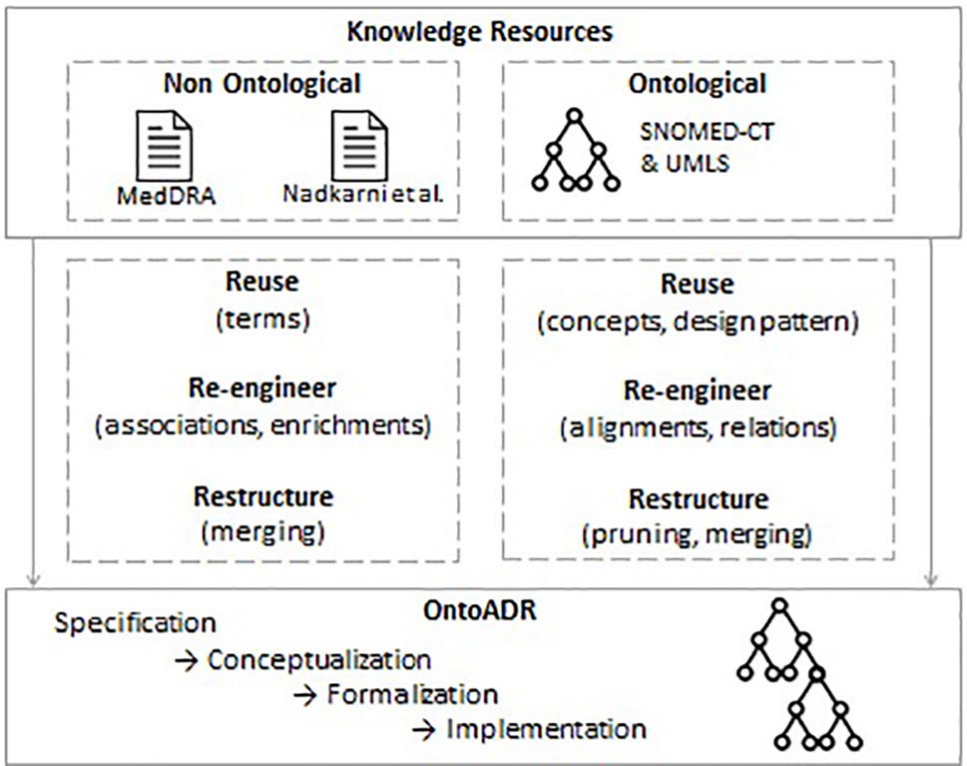
Scenarios of the NeON methodology for implementing OntoADR.

We applied the following scenarios of the NeON methodology for implementing OntoADR (**Figure 5**).

- Scenario 1. From Specification to Implementation: this includes four steps (specification, conceptualization, formalization, and implementation). We previously presented these steps in previous work [(Bousquet et al., 2014)] and limit here the scope of this presentation to scenarios that emphasize the reuse of ontological and non-ontological resources.
- Scenario 2. Reusing and Re-engineering Non-Ontological Resources: We transformed MedDRA into a subsumption tree (see [(Bousquet et al., 2014)], and retrieved a non-ontological resource provided by Nadkarni & Darer (2010). We analyzed this non-ontological resource in order to establish the correspondence between its content (mappings between MedDRA terms and SNOMED CT) and formal definitions that benefit from explicit relations derived from the SNOMED CT concept model. Then, we generated the formal definitions based on this non-ontological resource for inclusion in OntoADR.
- Scenario 6. Reusing, Merging, and Re-engineering Ontological Resources: We reused available ontological resources (UMLS, SNOMED CT) and merged SNOMED CT with MedDRA using mappings available in UMLS. We also reused the SNOMED CT concept model and had to reengineer it to keep only classes and relations that are relevant to formally define MedDRA terms. While the concept model may be considered as a building block that enforces best practices for ontological design in SNOMED CT, it does not strictly correspond to the description of content ontology design patterns, which explains why we did not implement Scenario 7: Reusing ontology design patterns (ODPs).
- Scenario 8. Restructuring Ontological Resources: This consists in pruning parts of the SNOMED CT that are not relevant for the description that was previously described by Souvignet et al., (2016b) and enriching the ontology by adding supplementary concepts and axioms.

In addition to these different scenarios, we also implemented “ontology support activities” for “knowledge acquisition” that comprises activities for (1) capturing knowledge from the MedDRA labels and work by a domain expert for adding formal definition using the Ci4SeR tool [(Souvignet et al., 2014)] and (2) “ontology validation” that consists in checking that the meaning of the ontology definitions are compliant with the definitions we intended the MedDRA terms to convey.

#### Flow chart of the method

**Figure 6:**
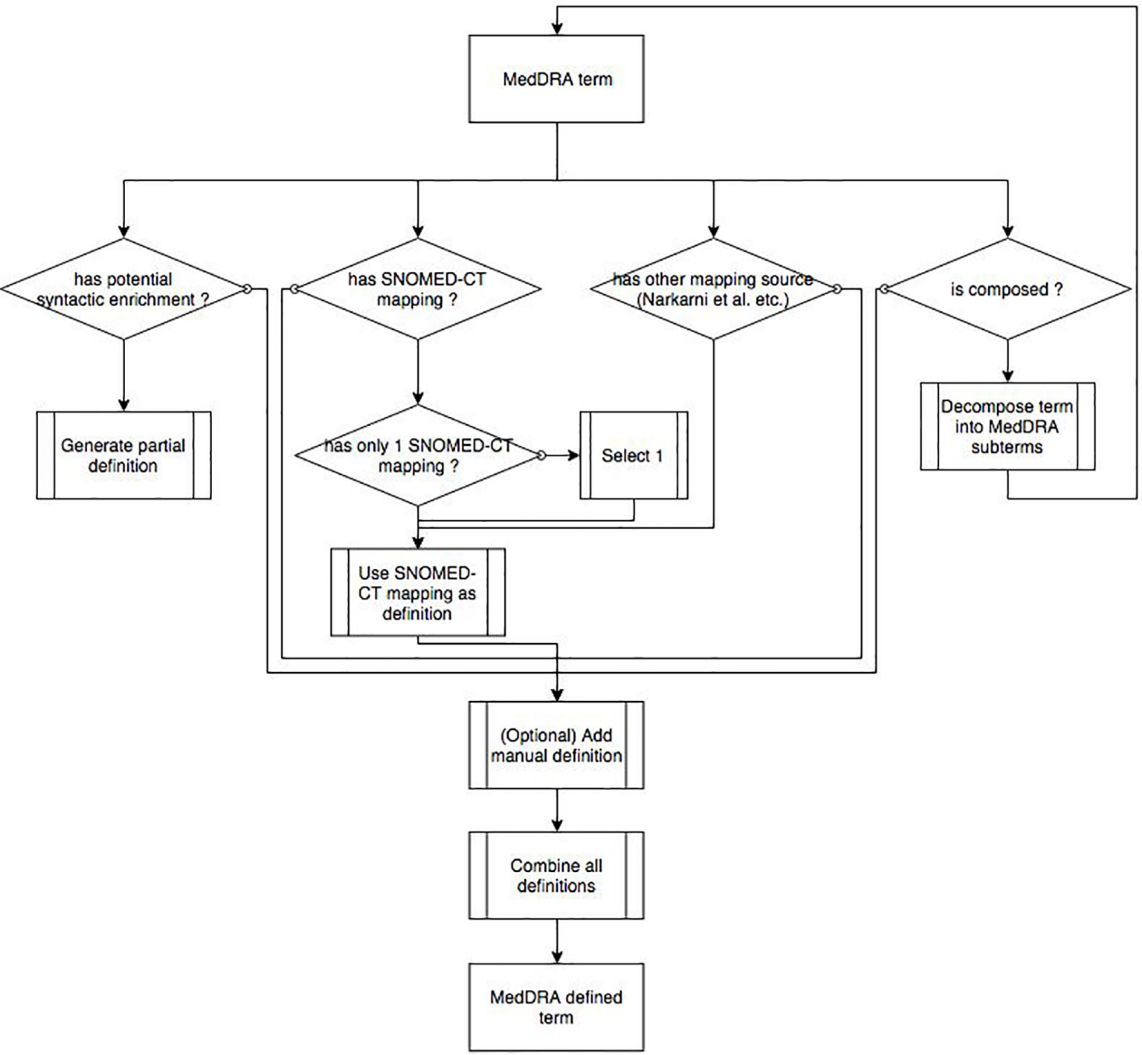
Flow chart representing an overall representation of the several steps and tasks.

**Figure 6** depicts a flow chart representing an overall representation of the several steps and tasks proposed in the article to get an overview of the algorithm at a glance. While this diagram could make readers believe that all these steps were conducted in parallel, it is proposed only as a convenient way to apprehend the method as a whole. The previous paragraph where we applied the NeON methodology shows a different perspective where different scenarios were applied at different time. In the flow chart, each MedDRA term is considered one after the other and can go through several parallel paths according to different conditions.

- If a MedDRA term is associated to a SNOMED CT concept in the UMLS metathesaurus, one mapping is manually selected: the use of the mapping information is described in the section *Using MedDRA-to-SNOMED CT Mappings From UMLS Metathesaurus*.
- If a MedDRA term is present in another mapping source than the UMLS (e.g., the Nadkarni and Darer’s proposal), then this mapping is used for the definition in a way that is described in the following section: *Using Another MedDRA-to-SNOMED CT Mapping Resource*.
- If a MedDRA term is composed of several distinct terms by a conjunction (e.g., “acute and chronic thyroiditis” that consists of two medical conditions “acute thyroiditis” and “chronic thyroiditis”), then the MedDRA term is decomposed according to the algorithm described in the section *Using a Syntactic Decomposition Algorithm on Complex MedDRA Terms*. Then, MedDRA subterms are considered as additional MedDRA terms that can be used as a new input for the whole algorithm.
- If a MedDRA term contains a substring with associated meaning (e.g., medical words ending in -algia that indicate pain), this MedDRA term may benefit from a potential syntactic enrichment: the generation of a partial definition is described in the section *Automatic Lexical Enrichment Methods*.

Some manual definitions can optionally be added (see the section *NeON Methodology*). All partial definitions acquired with the different algorithms are then automatically combined into a merged definition of the MedDRA term. We used MedDRA version 17 that consists of 26 SOC, 334 HLGT, 1,720 HLT, 20.559 PT, and 72,637 LLT, SNOMED CT version March 2015 and UMLS version 2014AB. SNOMED CT concepts were extracted from the Concepts_Core_INT file (Release Format 1) and the hierarchy and semantic properties from the Relationships_Core_INT file. This version of MedDRA was applied to the following paths: “using UMLS metathesaurus mappings,” “automatic enrichment methods,” and “manual definition of concepts.” MedDRA 13 that consists of 26 SOC, 335 HLGT, 1,709 HLT, 18,786 PT, and 68,258 LLT was applied to the following paths: “Using other mapping resources” and “Using a decomposition algorithm and Metamap software to map complex MedDRA terms.”

#### Problems With Mapping Other Layers Than the PT Level

We have tried to map other layers (SOC, HLT, and HLGT). For instance, the cardiac disorders SOC concept has for formal definition hasFindingSite some “Heart Structure.” While some SOC, HLGT, and HLT were accurately defined, essentially thanks to mappings in UMLS, it was decided not to present the results in this article. We used formal definitions associated with the three MedDRA higher levels only in the Ci4SeR tool (Souvignet et al., 2014), where the curator could use them if she considered them as relevant. We decided not to map the LLT because PT is the preferred level for case report analysis and search in pharmacovigilance databases.

We explained in previous work why the MedDRA hierarchy cannot be converted into a subsumption tree because this sometimes causes semantic inconsistencies (Bousquet et al., 2014). The reason is that most high level categories in MedDRA are intended to reflect the domain actors’ practices (i.e., following the different medical specialties) and are not necessarily organized according to different semantic criteria as one would expect in a well-formed ontology. A first example concerns groups of symptoms (HLGT or HLT) that are placed under the general categories of disorders that they are the symptom of (SOC or HLGT). Such hierarchical organization would not be authorized in an ontology as the relation being-a-symptom-of does not imply an is-a relation. For instance, the PT “dyspnoea” and “dizziness or syncope” belong to the HLGT “cardiac disorder signs and symptoms” that is under the SOC cardiac disorders. While dyspnea for instance refers to conditions that may be associated to cardiac disorders, such symptom cannot be considered as a cardiac disorder. A second example is the MedDRA PT sudden death that belongs to the following hierarchies: 1) ventricular arrhythmias and cardiac arrest (HLT)/cardiac arrhythmias (HLGT), and 2) death and sudden death (HLT)/fatal outcomes (HLGT). While “cardiac arrhythmias” is defined in OntoADR with the hasFindingSite some “Heart Structure” property, the sudden death PT should not inherit from such property because sudden death could be the consequence of death that is not of cardiac origin.

### Using Medical Dictionary for Regulatory Activities-to-SNOMED Clinical Terms Mappings From UMLS Metathesaurus

The UMLS may be used as a source of knowledge for adding formal definitions to medical terminologies (Schulz and Hahn, 2001). Based on our initial experience (Alecu et al., 2008), we assume that SNOMED CT is currently the best candidate for providing formal definitions to MedDRA. SNOMED CT terms are defined using description logic (DL) formalism, and a fair number of alignments between MedDRA and SNOMED CT are present in the UMLS metathesaurus. Therefore, most of the formal definitions attributed to MedDRA terms in OntoADR are based on semantic information extracted from the SNOMED CT clinical terminology. When a MedDRA term is mapped to a SNOMED CT concept, we reused the semantic information within SNOMED CT in order to build the formal definition of the MedDRA term. Identifying reliable MedDRA-to-SNOMED CT mappings is thus an essential step in our methodology to define MedDRA term semantics.

The UMLS (Lindberg et al., 1993) consists of a semantic network and a metathesaurus developed by the US National Library of Medicine to link terms from more than a hundred controlled vocabularies, including SNOMED CT and MedDRA. Terms from the different vocabularies are linked together by association to a unique UMLS concept defined by a concept unique identifier, e.g., “C0019163” that is mapped to both MedDRA term “hepatitis B” and SNOMED CT concept “type B viral hepatitis.”

In OntoADR, MedDRA term “hepatitis B” has the formal definition: hasFindingSite some “Liver Structure,” hasAssociatedMorphology some “Inflammation Morphology,” and hasCausativeAgent some “Hepatitis B Virus” where has FindingSite, hasAssociatedMorphology, and hasCausativeAgent are OntoADR semantic relations inspired from SNOMED CT, and “liver structure,” “inflammation morphology,” and “hepatitis B virus” are SNOMED CT concepts we imported in OntoADR (**Figure 1**).

In order to map MedDRA and SNOMED CT terms, we developed an algorithm following these steps: i) search for the MedDRA PT in the UMLS using the MedDRA identifier; ii) for MedDRA PT without SNOMED CT mappings in the UMLS, if the PT has one or more related LLT considered as synonymous, then the LLT identifier is used for a new UMLS search. iii) If this second search is unsuccessful, the algorithm performs a last UMLS search using PT and LLT labels, seeking to pair these labels with SNOMED CT concepts by string matching.

All mapping propositions selected from the UMLS metathesaurus were validated, modified, or completed by knowledge engineers and pharmacovigilance experts of our team. i) All one-to-one mappings we decided to use were first validated by checking manually the correspondence between the meanings of terms.

Several SNOMED CT concepts may be proposed as synonyms (Fung et al., 2005) of the same MedDRA concept, although they have different meanings. Each MedDRA concept in OntoADR can have only one equivalent SNOMED CT concept. When several SNOMED CT concepts are proposed in UMLS as synonym of a MedDRA term, only one was selected by an expert for building the formal definition. Such selection should be based on synonymy between a SNOMED CT concept and a MedDRA term. According to Fung, synonymy between term X and Y may be defined according to linguistic criteria (Fung et al., 2005) such as enforcing that it is possible to replace X by Y in any sentence without modifying the meaning. Examples of such synonyms are “celiac disease” and “gluten enteropathy,” or “kidney stone” and “renal calculus.” Selection of a SNOMED CT concept was performed first by comparing its label with the MedDRA term label. In case both were identical, which occurred most of the time, it was obvious to select this mapping, but in other cases, we took into account the medical relevance of the mapping and had to rely on expert evaluation. For example, three SNOMED CT concepts are mapped to the MedDRA term “Spondylitis”: “inflammatory spondylopathy,” “undifferentiated spondylitis,” and “spondylitis,” and the later appears as a perfect match according to label comparison.

Such a validation process was necessary because UMLS mapping propositions are not always semantically valid. MedDRA terms and SNOMED CT concepts mapped together in UMLS can refer to different medical entities, even if they are homonyms. For instance, the MedDRA term “vascular disorders” and its SNOMED CT homonym “vascular disorder” are mapped together in UMLS; however, the former refers to disorders of blood and lymphatic systems and the later only to disorders of blood vessels (lymphatic system disorders are caught by the concept “disorder of lymphatic system” in SNOMED CT). ii) In case of one-to-n mappings, a manual expert choice was made to select the SNOMED CT concept whose definition best fitted the meaning of the correspondent MedDRA concept. iii) When no SNOMED CT concept among the ones suggested by UMLS was satisfactory, the definition of the correspondent MedDRA concept was made manually. iv) Mapping a MedDRA term with a SNOMED CT concept does not ensure that the former gets a complete (or even a satisfying) formal definition of its semantics: the formal definition can be incomplete or even be literally absent: it is common to find SNOMED CT concepts, for instance psychiatric concepts, that have no definitional properties. When necessary, the semantic properties from SNOMED CT attributed to MedDRA concepts through the mapping procedure were thus completed manually by additional assertions.

### Using Another Medical Dictionary for Regulatory Activities-to-SNOMED Clinical Terms Mapping Resources

To complete the mappings selected from UMLS, we also made use of Nadkarni and Darer’s propositions of mappings (Nadkarni and Darer, 2010). Using one year of data (recorded between July 1, 2008 and April 30, 2009) from the US Food and Drug Administration Adverse Events Reporting System (AERS) pharmacovigilance database, the authors identified 3,705 MedDRA PT that collectively accounted for 95% of case reports. The 3,705 selected MedDRA terms correspond to high-frequency terms in the US Food and Drug Administration database and potentially have a great added value. After eliminating terms already mapped to SNOMED CT concepts in UMLS, they attempted to map manually the remaining terms (786 in total) with software assistance. Most of those terms (733) could be mapped by Nadkarni and Darer with SNOMED CT concepts *via* one-to-one or one-to-n mappings.

Several problems have been encountered when trying to reuse Nadkarni and Darer’s propositions of mappings (Nadkarni and Darer, 2010). i) First, in the case of one-to-n mappings, the authors broke down a MedDRA term in such a way to associate it to several SNOMED CT concepts but did not specify which semantic relation was relevant. For example, they mapped the MedDRA concept “tongue discoloration” with SNOMED CT concepts “abnormal color” and “entire tongue” but did not specify which semantic relation interconnected the first to the others. Obviously, it cannot be here an equivalence or synonymy (Fung et al., 2005) (“same as”) relation as in the case of one-to-one mapping. When SNOMED CT concepts belong to branches such as *body structure* or *morphologic abnormality*, the relationship to use is easy to deduce, and its creation can be automated: it will be in the first case the hasFindingSite relationship and, in the second, the hasAssociatedMorphology relationship. However, when it comes to SNOMED CT concepts from branches *finding*, *qualifier value*, or *disorder*, the relationship to use is not obvious, and only a human expert can decide. A major part of our recovery work was to specify these relationships by making use of the set of relationships available in OntoADR.

ii) We have also occasionally been forced to revise the proposed Nadkarni and Darer’s mappings, partly for reasons of pure semantic accuracy and partly because of the purpose of OntoADR, which we illustrate here using three examples:

- For the MedDRA term “feeding disorder neonatal,” the authors propose a mapping with SNOMED CT concepts “feeding disorder of infancy OR early childhood” and “neonatal.” The second mapping is correct, and we have included “neonatal” in OntoADR to define the MedDRA concept “feeding disorder neonatal” with the hasOccurrence relationship (hasOccurrence some Neonatal). We did not, however, reuse the first mapping. The concept “feeding disorder of infancy OR early childhood” is broader than the MedDRA concept “feeding disorder neonatal,” which concerns only the newborn. A mapping with SNOMED CT concept “feeding problems in newborn” would have been more accurate. To complete the formal definition of “feeding disorder neonatal,” we added the properties interprets some “Feeding pattern” and hasInterpretation some “Abnormal.”
- The MedDRA concept “azotaemia” is mapped by Nadkarni and Darer to SNOMED CT concepts “blood urea nitrogen measurement” and “increased.” We used the first by giving the MedDRA concept “azotaemia” the property interprets some Blood urea nitrogen measurement in OntoADR. However, the use of the second to describe the relationship hasInterpretation appeared problematic. Indeed, azotemia is characterized not so much by an increased concentration of nitrogen compounds in the blood but as a concentration above a certain reference threshold. We thus opted for the creation of the property hasInterpretation some Above reference range, more accurate in this context.
- For the MedDRA term “anorectal discomfort,” the authors propose a mapping with SNOMED CT concepts “discomfort” and “anus and rectum (combined site).” However, the problem here is that the SNOMED CT concept “anus and rectum (combined site)” is set in an isolated portion of the SNOMED CT branch “body structure” (branch called “group of anatomical entities”). Nothing connects it to the concepts of the digestive system structures (e.g., no relationship part-of). Due to the SEP decomposition (structure, entire, part) of the anatomical branch of SNOMED CT, the concept “anal structure” has no relation to the concept “anus and rectum.” It would have been impossible to use this localization by semantic reasoning, for example, to identify concepts located on part of the anorectal system (principle of subsumption reasoning: concepts that have a relationship of location on parts of the anorectal structure are considered by inference as siblings of the concept of diseases that are located on the whole anorectal structure). We therefore preferred to use the SNOMED CT concept “anorectal structure” to define the relationship hasFindingSite of the MedDRA concept “anorectal discomfort” in OntoADR. This SNOMED CT concept allows the semantic reasoning operation described previously. Moreover, we can assume that the MedDRA term “anorectal discomfort” is sometimes used to encode ADRs in a non-specified way that may be anal or rectal, and not both, as is implied by the use of the SNOMED CT concept “anus and rectum (combined site).” It is therefore important to locate by subsumption concepts that are located within a substructure of the whole anorectal structure.

### Using a Syntactic Decomposition Algorithm on Complex Medical Dictionary for Regulatory Activities Terms

Among MedDRA terms that are not mapped with SNOMED CT terms in UMLS, there are many complex terms, i.e., corresponding to composed expressions. MedDRA complex terms are of several kinds: a) expressions composed with an AND logical operator or commas (e.g., “acute and chronic thyroiditis” or “pregnancy, labour, delivery, and postpartum conditions”); b) expressions composed with “NEC” (not elsewhere classified), “unspecified,” or with a text between brackets, usually to specify exclusion clauses [e.g., “autoimmune disorders NEC,” “laryngeal neoplasms malignancy unspecified,” or “ocular neoplasms malignant (excl. melanomas)”]; c) they can also combine these different kinds of complexity [e.g., “gastrointestinal and abdominal pains (excl. oral and throat)” or “ocular structural change, deposit, and degeneration NEC”]. These terms are usually terms of level HLT, HLGT, and SOC in the MedDRA hierarchy. However, their definitions have a great added value because some terms they subsume may inherit their properties. Indeed, defining one high level term with a morphology property may amount to defining all child terms with this property within the limits of what we have indicated in the section *Problems With Mapping Other Layers Than the PT Level*.

The complex MedDRA terms present two kinds of difficulties: i) the difficulty of mapping with SNOMED CT that tends to favor simple concepts probably because most complex concepts correspond to pure classifying artifacts, e.g., “not elsewhere classified,” without real counterpart in the phenomena that are part of medicine; ii) difficulties for formalization of meaning: representing in OWL (Web Ontology Language) the meaning of a compound concept containing exclusions with logical operators is constrained by the expressiveness of the DL language used. In OntoADR, it is not possible to describe the exact same MedDRA semantics due to computability constraints. To date, we have developed a technical solution for the first point, but no satisfactory solution of conceptualization (especially in terms of human cost modeling) has yet been developed to meet the second point. It should be noted that this issue is regarding mainly terms of high levels and does not affect the progress of definitions for PT terms in OntoADR.

In order to map complex MedDRA terms, we developed an algorithm for syntactic decomposition. It consists of three routines: 1) a routine for “cleaning” terms; 2) a routine for identification of complex expressions; and 3) a routine for decomposition of an expression from a set of formal rules. Routine 1 begins by suppressing from the MedDRA labels unnecessary characters or characters that cannot be supported by the decomposition routine [stop words, content between brackets, terms as “unspecified,” “NOS” (not otherwise specified), etc.]. Routine 2 identifies decomposable expressions: it searches for keywords that indicate a probable composition of the expression (“AND,” “OR,” “WITH,” “,”, etc.). Finally, routine 3 decomposes the complex expression in a set of simpler expressions (cf. **Table 2**), by applying different rules, for example:

**Table 2:** Examples of parsing of complex MedDRA terms using different rules.

Input : MedDRA complex term	Selected Rule	Output : result of the decomposition
“Ear and labyrinth disorders”	(A AND B).q	“ear disorders” “labyrinth disorders”
“Manic and bipolar mood disorders and disturbances”	((A AND B).q).(C AND D)	“manic mood disorders” “bipolar mood disorders” “manic mood disturbances” “bipolar mood disturbances”
“Blood and lymphatic system disorders”	(A AND B).q	“blood system disorders” “lymphatic system disorders”

$$\begin{array}{c} (\text{A AND B}).\text{q}\to \text{ A}.\text{q}+\text{B}.\text{q}\\ \text{q}.(\text{A AND B})\to \text{ q}.\text{A}+\text{q}.\text{B} \end{array}$$

We then used the MetaMap software (Aronson, 2001) to map all new decomposed concepts to existing SNOMED CT concepts.

### Automatic Lexical Enrichment Methods

We have used a rule-based algorithm for automatic suggestion of properties from the MedDRA label to enrich the formal definition of concepts. Two key procedures have been implemented in the algorithm:

1. When the algorithm detects a given string S_x _in a MedDRA label, it automatically adds a corresponding property P_x_ in the OWL concept definition. For example: if the string “pain” or “algia” is found in a MedDRA concept’s label, the semantic property hasDefinitionalManifestation some Pain is automatically added to the concept’s definition. Similarly, if the string “perforation” is found, the formal definition hasAssociatedMorphology some Perforation is suggested. All created properties are then validated by an expert. Illegitimate properties are rejected. For example, the algorithm proposed to add the formal property hasAssociatedMorphology some Hernia to the MedDRA concept “hernia repair,” as the string “hernia” was found in the label. Semantically, this assignment is obviously illegitimate: the “hernia repair” is not a type of hernia and cannot be defined by this morphological property. The property has therefore been rejected. The expert, however, took advantage of this suggestion to correct it in: OccursAfter some Hernia. This validation step is also necessary due to the occasionally polysemic expressions used for automatic generation of properties. For example, the automatic generation of the hasClinicalCourse some Cyclic property, when the algorithm detects the “cyclic” string in a MedDRA label is valid for concepts such as “cyclic neutropenia” or “cyclic vomiting syndrome,” where the term “cyclic” indicates the clinical course of the disease. However, it is not valid for the concept “cyclic AMP,” which refers to a clinical test (a measure of the presence or amount of cyclic adenosine monophosphate, e.g., in urine). We could have improved the automatic processing in order to detect these problematic cases, but the formalization of these exceptions would have taken longer time than using a manual approval process.

A restriction is applied to prevent the property P_x_ to be duplicated when it already exists in a MedDRA term definition, e.g., the detection of the “perforation” string in the label of a MedDRA concept C_Med_ only results in the creation of the property hasAssociatedMorphology some Perforation if C_Med_ does not already own a property hasAssociatedMorphology some <Morphology>. If it is the case, we assume that the relation R_x_ (in this case hasAssociatedMorphology) has already been filled in correctly.

2. A second procedure is implemented by the algorithm to automatically generate properties. Based on the same principles, but working with more complex patterns of recognition, it was designed to complete definitions of MedDRA concepts referring to investigations and their results (SOC « Investigations »).

Two relationships are available in SNOMED CT to define the examination results (whether clinical observations or investigations): interprets, which refers to “the entity being evaluated or interpreted, when an evaluation, interpretation, or “judgment” is intrinsic to the meaning of a concept”; and hasInterpretation, which, grouped with the attribute Interprets, “designates the judgment aspect being evaluated or interpreted for a concept (e.g., presence, absence, degree, normality, abnormality, etc.)” (Rector and Brandt, 2008). It is important that these two relationships are filled in OntoADR in order to apply semantic reasoning not only to ADR concepts as such, but also to concepts referring to abnormal results of investigations that are the consequence of an ADR (for instance, “neutrophil count decreased” for the neutropenia condition), as such results are frequently used to describe ADRs in pharmacovigilance databases. However, it turned out that very few MedDRA concepts located in the investigations branch could be identified through the procedures described in the previous sections, in particular the mapping from UMLS. A large majority of MedDRA concepts in SOC investigations thus remained undefined in OntoADR.

To remedy this situation, we have integrated into the algorithm a module supporting the properties interprets and hasInterpretation for MedDRA concepts from SOC “investigations.” Results of investigations are usually expressed in MedDRA using the following adjectives: abnormal, normal, absent, present, increased, decreased, positive, and negative. All these qualifiers are also used in SNOMED CT to fill the property hasInterpretation. The procedure followed by the algorithm was therefore as follows:

When the string S_x_ corresponding to one of these adjectives is detected in the label < lab1> of a MedDRA concept C_Med1_ from SOC “Investigations”:

1. Create in the definition of C_Med1_ the property hasInterpretation some S_x_
2. Find if it exists in the investigations branch a concept C_Med2_, whose label < lab2> corresponds to (< lab1> minus S_x_). If C_Med2_ exists, create in the definition of C_Med1_ the property interprets some C_Med2_. This second phase of the procedure is used to connect *via* the property interprets the results to the related investigations.

In the example of the concept C_Med1_ « Alpha hydroxybutyrate dehydrogenase decreased », this procedure gives the following results:

1. Creation of the property hasInterpretation some Decreased in the definition of C_Med1_
2. There is a concept C_Med2_: “alpha hydroxybutyrate dehydrogenase”. The property interprets some ‘Alpha hydroxybutyrate dehydrogenase’ is thus created in the definition of C_Med1_.

Once again, all of the created properties were reviewed and validated by an expert.

### Manual Definition

Besides these semiautomatic methods for defining MedDRA concepts in OntoADR, we also performed the manual definition of about 1,935 concepts (Souvignet et al., 2016b). We had insufficient human resources to carry out the manual definition of all MedDRA terms that previous methods had failed to define. So, we decided to focus on high value-added terms for pharmacovigilance. In the EU-ADR project, Trifirò et al. (2009)developed a ranked list of 23 first importance adverse drug events (e.g., cardiac valve fibrosis) based on a review of scientific literature, medical textbooks, and websites of regulatory agencies. To identify which MedDRA terms are related to those 23 topics, pharmacovigilance experts familiar with MedDRA have chosen for each topic an SMQ and/or MedDRA hierarchy-based grouping (HLT or HLGT) or a custom set of preferred terms (PT) fitting the definition of the targeted topics (see Declerck et al., 2012 for details). When no existing MedDRA groupings could be identified to fit the safety topic, *ad hoc* manual groupings of MedDRA PT were proposed by the experts. This work benefited from using a dedicated tool we implemented, Ci4SeR (curation interface for semantic resources) (Souvignet et al., 2014).

## Results

### Using Other Medical Dictionary for Regulatory Activities-to-SNOMED Clinical Terms Mapping Resources

“Once the Nadkarni and Darer’s mapping propositions were validated, modified or completed, we applied the same procedure as described in the section *Using MedDRA-to-SNOMED CT Mappings From UMLS Metathesaurus* to pick up information from SNOMED CT and define the MedDRA concepts of OntoADR. Using the set of SNOMED CT relations available in OntoADR, we also realized manually the definition of those MedDRA terms (53 in total) for which no mapping could be found by Nadkarni and Darer. The use, after verification and eventually correction and complementation, of mappings proposed by Nadkarni and Darer, allowed us to complete the definition of 786 supplementary MedDRA PTs in OntoADR.

### Using a Syntactic Decomposition Algorithm on Complex Medical Dictionary for Regulatory Activities Terms

Among the 2,070 HLT, HLGT, and SOC in MedDRA 13.0, a total of 1,011 terms was decomposed by the algorithm generating an average of 2.7 terms by decomposition. The consistency of automatic decomposition was checked by an expert. The errors were corrected through a progressive adjustment of the decomposition algorithm. Only the decomposition of 30 complex terms that were not supported by the algorithm was done manually. Once the decomposition was performed, we used the UMLS MetaMap 2010 AB mapping software, which returns from a given string (in our case, a part of the decomposition), the UMLS concept unique identifier of the nearest syntactically SNOMED CT concepts (fuzzy match). With this method, a total of 638 MedDRA concepts (9 SOCs, 131 HLGTs, and 498 HLTs) could be mapped to the SNOMED CT concepts (mappings one-to-one or one-to-n).

This additional mapping method has the advantage of enabling the definition of high level terms in MedDRA. These definitions may then be inherited by subsumed low level terms. However, the definitions have also the disadvantage of being broad and thus potentially insufficiently precise for specific preferred terms.

### Automatic Lexical Enrichment Methods

This procedure was applied to 11 of the 25 SNOMED CT properties used in OntoADR, using 82 different matching strings. In total, this procedure has led to the creation of 8,194 properties, among which 7,691 were validated (i.e., 93.9%). A sample of the strings detected by the algorithm and properties created is shown in **Table 3**.

**Table 3:** Sample of the properties created automatically from the MedDRA label to enrich the formal definitions of MedDRA concepts in OntoADR.

Relation	Matching strings	Value of the property	Nb properties created	% properties validated
**hasClinicalCourse**	acute cyclic recurrent	Sudden onset and/or short duration Cyclic Recurrent	84 10 121	95.1% 20% 100%
**hasCausativeAgent**	bacteria viral	Bacteria Virus	83 157	100% 88.5%
**hasAssociatedMorphology**	abscess hernia haemorrhage, haemorrhagic, bleeding	Abscess morphology Hernia Hemorrhage	121 70 272	100% 82.9% 86.4%
**hasPathologicalProcess**	infection, infections, infectious, infective parasitic	Infectious process Parasitic process	726 12	89.4% 91.7%
Interprets	motor, movement, kines	Motor function behaviour	66	59.1%
DueTo	allergic	Hypersensitive reaction	32	93.8%

### Manual Definition of Concepts

**Figure 7:**
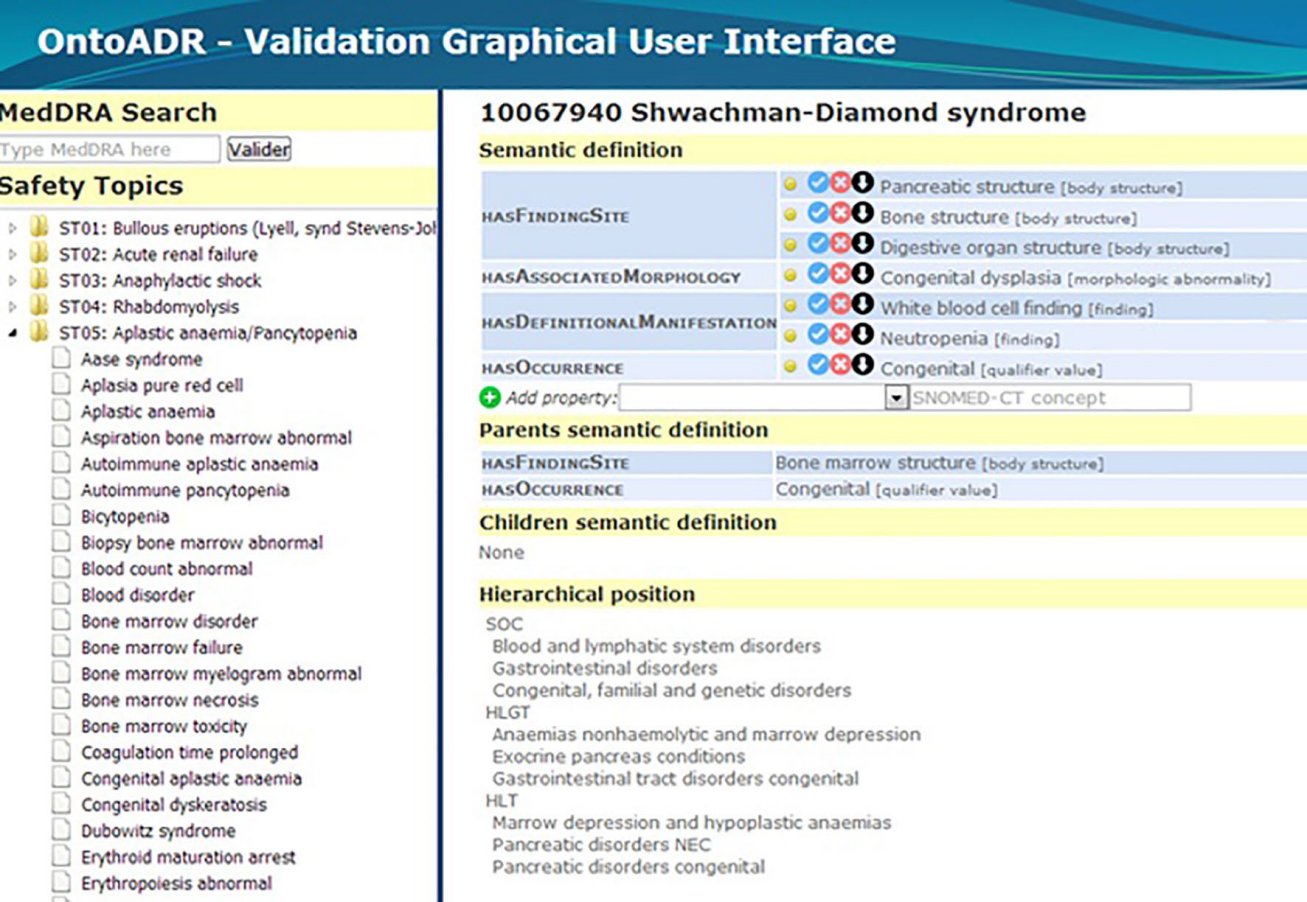
Formal definition associated to the preferred term “Shwachman-Diamond syndrome” before manual refinement.

**Figure 7** depicts as an example the formal definition associated to the term “Shwachman-Diamond syndrome,” as it was described in OntoADR after application of the different algorithms that precede manual refinement.

The curation, which took approximately 750 h, allowed refining the definition of 1,935 MedDRA terms to validate and fully define these terms (Souvignet et al., 2016b). Among the 3,482 properties available in OntoADR for these terms, the curator validated 2,636 properties (76%), proposed 350 (10%) more precise terms (i.e., narrower terms in the SNOMED CT hierarchy), and removed 496 properties (14%). The curator also proposed 13,675 additional properties, but these should not be considered as errors related to missing properties but rather as the curator’s desire to better document diagnoses with signs and symptoms and investigations that may be associated to a given disease but are not specific, as they may be absent in some occurrences of this disease.

**Figure 8:**
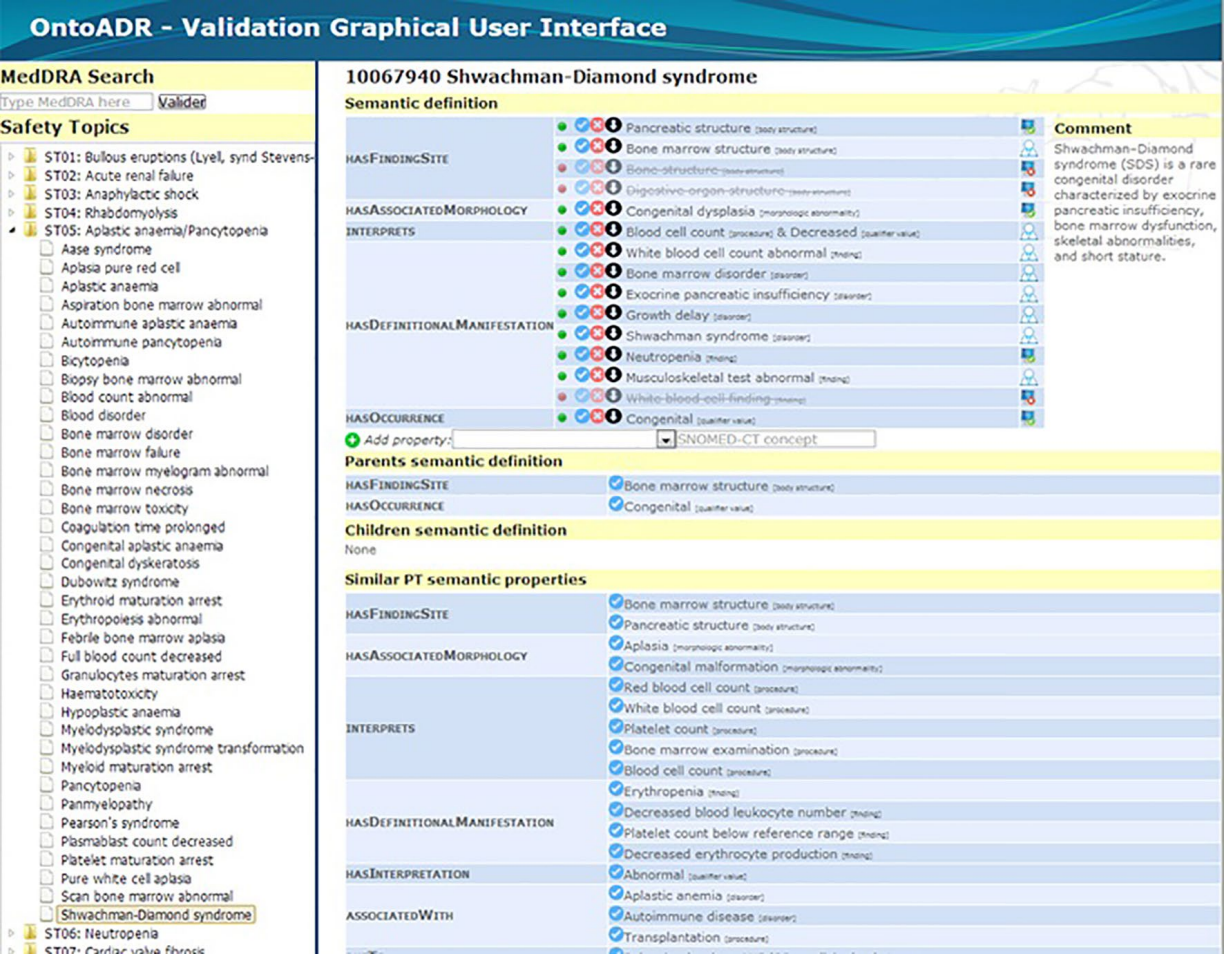
Formal definition associated to the term “Shwachman-Diamond syndrome” after manual refinement.

**Figure 8** shows how the “Shwachman-Diamond syndrome” PT’s formal definition was modified by the curator in the Ci4SeR tool. The lowest part of the screenshot contains the properties that were automatically proposed considering the parent’s and siblings’ formal definitions. **Table 4** depicts the results using each method.

**Table 4:** Synthesis of mappings and properties found using all previously described methods.

Source/Method	MedDRA version	Comparator	Number of mappings	Number of properties
Using UMLS Metathesaurus mappings	v17	20,599 PT	11,281 PT (54.8%)	74,598
Using other mapping resources	v13	18,786 PT	455^a^ PT (2.4%)	469^a^
Using a decomposition algorithm and Metamap software to map complex MedDRA terms	v13	2,070 HLT, HLGT, and SOC	638^a,b^ HLT, HLGT, and SOC (30.8%)	*–*
Automatic enrichment methods	v17	*–*	*–*	7,691^a^
Manual definition of concepts	v17	20,599 PT	1,935 PT (9.1%)	13,675

^a^Represent only the number of mapping/properties that was not found by other methods.

^b^Limited to SOC, HLGT, and HLT.

## Discussion

### Summary

We have described in this article several methods that allow collectively a better semantic enrichment of MedDRA. **Table 4** shows that using UMLS metathesaurus is the method that was the most efficient considering the number of mappings and helped to add formal definitions for about half MedDRA terms. As other mapping resources than UMLS are rare and concern only few MedDRA terms, the Nadkarni and Darer’s resource allowed to add properties to 4.2% of MedDRA terms but only to 2.4% of MedDRA terms that were not associated with mappings to SNOMED CT in UMLS.

Our proposal to decompose complex MedDRA terms was applied only to SOC, HLGT, and HLT levels and accounted for 30.8% of these MedDRA terms above the PT level. Manual definitions and refinements of definitions obtained with other methods allowed to process 9.1% of MedDRA terms, which is more than the proportion of terms that were defined using Nadkarni and Darer’s mapping resource. However, it was associated with high time-consuming effort by the domain expert that confirms previous work, e.g., Giannangelo and Millar (2012) who observed that “map specialists on average mapped 6.5 SNOMED CT concepts an hour.” **Table 5** summarizes the main characteristics of each method and indicates if the proposed method reuses existing knowledge, if it requires manual adaptations or may be performed in an automated way.

**Table 5:** Summary of inconveniences and advantages of the different methods.

Algorithms	What is already available?	Characteristics of the algorithm
Using MedDRA-to-SNOMED CT mappings from UMLS Metathesaurus	MedDRA-to-SNOMED CT mappings in UMLS Metathesaurus	Available mappings are used to retrieve SNOMED CT concepts associated to a MedDRA term. Properties are added to the formal definition according to the SNOMED CT concept position in the hierarchy. It illustrates Scenario 6. “Reusing, Merging and Re-engineering Ontological Resources” of the NeON methodology. The algorithm is automatic, except when several SNOMED CT concepts are available which requires expert selection.
Using other MedDRA-to-SNOMED CT mapping resources	Nadkarni and Darer’s propositions of mappings	This is an expert-based process entirely manual of validation and refinement that illustrates Scenario 2. “Reusing and Re-engineering Non-Ontological Resources” of the NeON methodology but benefits from a non-ontological resource that expedites formal definition of MedDRA terms compared with manual definitions.
Using a syntactic decomposition algorithm on complex MedDRA terms	*–*	This algorithm is automated and developed ad hoc. It illustrates one of the ontology support activities, “knowledge acquisition,” and exploits hidden semantics as proposed by Third (2012). It is limited to complex MedDRA terms.
Automatic lexical enrichment methods	*–*	This algorithm is based on substring search. It necessitates defining beforehand substrings that may be associated to a SNOMED CT concept. Review of the algorithm’s proposal is mandatory in order to check that substrings allow associating with relevant SNOMED CT concepts. It also illustrates Ontology support activity “knowledge acquisition” and exploits hidden semantics.
Manual definition	*–*	This process is manual and expert based, but the Ci4SeR tool suggests definitions on the basis of definitions already available for siblings and parents of a MedDRA PT. Such approach addresses both “knowledge acquisition” and “ontology validation” within the Ontology support activities.

### Related Work in Medical Informatics

He et al. (2014) have introduced the Ontology of Adverse Events (OAE). OAE was originally targeted for vaccine adverse events (Marcos et al., 2013) and now also includes adverse drug events. In practice, using OAE to select case reports in the Vaccine Adverse Event Reporting System proved difficult: “AE data stored in Vaccine Adverse Event Reporting System are annotated using MedDRA” (Marcos et al., 2013). Authors complained that “many disadvantages of MedDRA, including the lack of term definitions and a well-defined hierarchical and logical structure, prevent its effective usage in VAE (vaccine adverse event) term classification.” Therefore, for an efficient analysis, they performed a mapping between MedDRA and OAE (Sarntivijai et al., 2012).

OAE contains about 2,300 AE entities but only 1,900 MedDRA mappings (9% of all MedDRA PT). For example, there is a single term for upper gastrointestinal hemorrhage in OAE (He et al., 2014), whereas one can cite several in MedDRA (see the section *Rationale for Supplementing MedDRA With Formal Definitions* where we identified 27 using OntoADR). Furthermore, OAE formal definitions are limited to anatomical and physiopathological descriptions. He and colleagues proposed extensions to OAE such as the Ontology of Drug Neuropathy Adverse Events (Guo et al., 2016), which suggests that providing supplementary MedDRA mappings is possible using the same methodology. One advantage of OAE is the possibility to use it in open access, which allows wide dissemination to users, while legal issues related to ownership of MedDRA and SNOMED CT should be solved before we can make OntoADR available.

Adverse Events Reporting Ontology aims to allow storing of pharmacovigilance data related to anaphylaxis according to guidelines defined by the Brighton collaboration (Courtot et al., 2014) but may also be extended to other safety topics, e.g., malaria (Courtot et al., 2013). Nevertheless, ADRs are not formally defined in Adverse Events Reporting Ontology.

While we did not find any resource available providing definitions for every ADR in MedDRA, there are more general resources with formal representation of clinical terms. In order not to start from scratch the definitions of ADRs, we needed a trustworthy formal resource, standardized and reliable. We chose SNOMED CT for three main reasons: first, pharmacovigilance concepts generally do not differ from those used in other medical ﬁelds. Second, SNOMED CT is the most complete and most detailed terminology of medicine with a formal semantic foundation currently available (Elkin et al., 2006) sharing common fields with MedDRA (medical pathologies in all medical specialties, signs and symptoms, laboratory tests results, some diagnostic and therapeutic procedures). Finally, SNOMED CT has the advantage of covering to a large extent, if not entirely, other standard medical terminologies such as International Classification of Diseases, 10^th^ edition (ICD-10), and especially more than 50% of MedDRA terms (excluding LLT) are associated with a SNOMED CT concept (Bodenreider, 2009) in UMLS, a degree of coverage that, to our knowledge, no other current medical ontology was able to match.

We found in the literature several examples of mappings from a terminology to SNOMED CT (Vikström et al., 2007; Merabti et al., 2009; Nyström et al., 2010; Dhombres and Bodenreider, 2016; Fung et al., 2017). However, the objective was usually to integrate a terminology in SNOMED CT or to map this terminology to SNOMED CT but not to enrich this terminology by the means of formal definitions. The lexically assign logically refine method is an example of an automated method in which logical observation identifiers names and codes (LOINC) and SNOMED terms are first decomposed, then refined by the means of knowledge-based methods that allowed to map LOINC and SNOMED together (Dolin et al., 1998). In another work, Adamusiak and Adamusiak and Bodenreider (2012) developed an OWL version of both LOINC and SNOMED CT and made use of mappings between SNOMED CT terms to identify redundancy and inconsistencies in LOINC multi-axial hierarchy. Roldán-García et al. (2016) implemented Dione, an OWL representation of ICD-10-CM where formal definitions were obtained thanks to mappings between ICD-10-CM and SNOMED CT available in UMLS and the Bioportal. More recently, Nikiema et al. (2017) benefited from SNOMED CT logical definitions to find mappings between ICD-10 and ICD-O3 concepts in the domain of cancer diagnosis terminologies.

It is usually recommended to build medical terminologies following the model of clinical terminologies that obey to Cimino’s desiderata (Cimino, 1998; Bales et al., 2006). Such model brings several advantages such as improving the maintenance of large terminologies (Cimino et al., 1994), and formal definitions were implemented in several terminologies such as the NCI-Thesaurus (Hartel et al., 2005). Our approach is more in line with what is recommended by Ingenerf and Giere (1998), that is to say, to keep terminologies with disjoint classes required for statistics (in a clinical terminology, the same term may be present in several separate categories because of multiple inheritance and be counted more than once) and instead implement a mapping of terms of first-generation system to a formal system. This allows keeping the MedDRA terminology in its current format, counting ADRs according to predefined categories that are standardized and replicable at the international level with MedDRA and building new categories on demand by using knowledge engineering methods. This is what we have done in our implementation of OntoADR (Bousquet et al., 2014) in the form of an OWL-DL file and in the form of a database (Souvignet et al., 2016b).

We have no knowledge of other works in which the formalization of complex terms involving AND/OR relations has been performed in an automated way. We have not proposed formal definitions of LLT because this level is reserved for the coding of case reports, in order to improve the accuracy of coding, but it is not useful for grouping data for analysis (which is performed at the PT level). Although the analysis of pharmacovigilance databases is performed preferentially at the PT level, it could be important to also define the upper levels: SOC, HLGT, and HLT. This formalization would bring several advantages: i) preferred terms may inherit properties from their parents that allows to give them a formal definition in case the synonymous SNOMED CT concept has no definition, or there is no SNOMED CT concept mapped to this PT in UMLS; ii) This would allow to calculate by the means of terminological reasoning high level MedDRA categories in which PTs should be included and therefore restore multiple inheritance that does not exist in MedDRA. However, it is advisable to remain modest insofar as the relations between a PT and the higher hierarchical levels to which it is attached are not always of a taxonomic nature.

### Perspectives

Our perspectives are to add formal definitions to a larger number of MedDRA terms. Our approach may be improved using more advanced natural language processing techniques (Iavindrasana et al., 2006; Deléger et al., 2009; Liu et al., 2011; Dupuch et al., 2014) compared with the basic semantic enrichment we performed considering MedDRA labels. We estimate that the methods proposed here can be reused for other first-generation terminologies provided that these terminologies have a mapping with SNOMED CT with fair coverage and that this mapping is available in accessible sources of knowledge such as the UMLS. The terminology can also be treated using methods of natural language processing as was done for example with LOINC in the lexically assign logically refine method (Dolin et al., 1998). One can also consider cases in which the terminology would be normally defined by mapping to another clinical terminology than SNOMED CT. This may be the case in other areas of application in which SNOMED CT is not the best choice.

As the manual approach was time consuming and necessitates human resources we do not have, we plan to rely on the development of complementary automated approaches. First, formal definitions could be extracted from textual definitions (Petrova et al., 2015) or directly using morphosemantic analysis on the term label, e.g., blepharitis where “itis” stands for “inflammation,” and “blephar” stands for “eyelid.” Such approach is limited to terms containing “compound forms” that have a medical meaning (Deléger et al., 2009). Second, formal definitions could be based on ontology design patterns, such as implemented in tools like Ontorat (Xiang et al., 2015) or TermGenie (Dietze et al., 2014), which partially automate the process, as they still rely on expert curation. Third, additional mappings between MedDRA and other terminologies could be obtained *via* improved mappings in the UMLS metathesaurus (Bodenreider et al., 1998; Fung et al., 2007; Diallo, 2014). Fourth, semantic definitions may be audited by comparing definitions associated to terms that present lexical similarities (Agrawal and Elhanan, 2014). However, this presents an intrinsic limit: terms to compare should consist of at least three words that constraints this method mainly to MedDRA procedures.

Fifth, we plan to extract knowledge using additional sources than SNOMED CT such as NCI Thesaurus (Sioutos et al., 2007) that could be useful to build definitions for MedDRA terms that describe cancer-related adverse reactions. A recent work by Oliveira and Pesquita, (2018) reports that current ontology matching techniques and systems are mostly devoted to finding links between two equivalent entities from two distinct ontologies. However, different domains may be involved that requires the implementation of matching techniques that allow linking more than two ontologies through more complex relations. An example is “aortic valve stenosis” (from human phenotype ontology) that is equivalent to the combination of “aortic valve” (from the Foundational Model of Anatomy) and “constricted” (from Phenotype And Trait Ontology).

## Conclusion

The possibility of selecting terms using formal definitions and terminological reasoning are major advantages of clinical terminologies with formal semantics such as SNOMED CT, which present several advantages compared with classic terminologies. MedDRA, as a standard international terminology for the coding of ADRs in pharmacovigilance databases, could beneficiate from these knowledge engineering techniques, but MedDRA terms have to be defined using formal languages first. As defining manually MedDRA terms takes much time, it is important to reuse as much as possible ontological and non-ontological resources available to expedite the generation of formal definitions. The collection of methods we present can collectively support a semiautomatic semantic enrichment of MedDRA. Perspectives are to implement more efficient techniques to find more logical relations between SNOMED CT and MedDRA in an automated way.

## Author Contributions

GD adapted Nadkarni and Darer’s definitions to OntoADR, performed automatic lexical enrichment methods, and wrote the first draft of the manuscript. M-CJ provided significant advice on the design of the study and contributed to the evaluation. ES first, then JS, performed mappings between MedDRA and SNOMED CT using the UMLS metathesaurus and developed new versions of OntoADR. JS performed the syntactic decomposition algorithm on complex MedDRA terms and wrote the corresponding section of the manuscript. CB conducted the study, contributed to the evaluation, reviewed state-of-the-art related work, and wrote the final article. CB and M-CJ were responsible for submitting the PROTECT project to the IMI requests for proposal and for submitting the PEGASE project to the ANR request for proposal. All authors have made substantial contributions and approved the final manuscript and agreed to be accountable for all aspects of the work.

## Funding

This work was funded by the grant N° ANR-16-CE23-0011-01 from the ANR, the French Agence nationale de la Recherche through the PEGASE (*Pharmacovigilance enrichie par des Groupements Améliorant la detection des Signaux Emergents*). Some results described in this article were obtained as part of the PROTECT consortium (Pharmacoepidemiological Research on Outcomes of Therapeutics by a European Consortium, www.imi-protect.eu) which is a public–private partnership coordinated by the European Medicines Agency. The PROTECT project has received support from the Innovative Medicine Initiative Joint Undertaking (www.imi.europa.eu) under grant agreement no. 115004, resources of which are composed of financial contribution from the European Union’s Seventh Framework Program (FP7/2007-2013) and EFPIA companies’ in kind contribution.

## Conflict of Interest Statement

The authors declare that the research was conducted in the absence of any commercial or financial relationships that could be construed as a potential conflict of interest.

## Abbreviations

ADR, Adverse drug reactions; AERS, Adverse Events Reporting System; Ci4SeR, Curation Interface for Semantic Resources; HLGT, Higher Level Group Term; HLT, High Level Term; ICD-10, International classification of diseases, 10th edition; LALR, Lexically assign logically refine; LLT, Lowest Level Term; LOINC, Logical Observation Identifiers Names & Codes; MedDRA, Medical dictionary for drug regulatory activities; NEC, Not elsewhere classified; NOS, Not otherwise specified; OAE, Ontology of Adverse Events; OWL, Web Ontology Language; PT, Preferred Term; SMQ, Standardized MedDRA Queries; SNOMED CT, SNOMED Clinical Terms; SOC, System Organ Class; UMLS, Unified Medical Language System; WHO ART, World Health Organization-Adverse Reaction Terminology.
